# Bioinformatics and system biology approach to identify the influences of SARS-CoV-2 on metabolic unhealthy obese patients

**DOI:** 10.3389/fmolb.2023.1274463

**Published:** 2023-10-09

**Authors:** Tengda Huang, Nan Jiang, Yujia Song, Hongyuan Pan, Ao Du, Bingxuan Yu, Xiaoquan Li, Jinyi He, Kefei Yuan, Zhen Wang

**Affiliations:** Division of Liver Surgery, Department of General Surgery and Laboratory of Liver Surgery, and State Key Laboratory of Biotherapy, West China Hospital, Sichuan University, Chengdu, China

**Keywords:** SARS-CoV-2, metabolic unhealthy obese, differentially expressed genes, gene ontology, protein-protein network (PPI), hub gene, drug molecule, gene-disease association

## Abstract

**Introduction:** The severe acute respiratory syndrome coronavirus 2 (SARS-COV-2) has posed a significant challenge to individuals’ health. Increasing evidence shows that patients with metabolic unhealthy obesity (MUO) and COVID-19 have severer complications and higher mortality rate. However, the molecular mechanisms underlying the association between MUO and COVID-19 are poorly understood.

**Methods:** We sought to reveal the relationship between MUO and COVID-19 using bioinformatics and systems biology analysis approaches. Here, two datasets (GSE196822 and GSE152991) were employed to extract differentially expressed genes (DEGs) to identify common hub genes, shared pathways, transcriptional regulatory networks, gene-disease relationship and candidate drugs.

**Results:** Based on the identified 65 common DEGs, the complement-related pathways and neutrophil degranulation-related functions are found to be mainly affected. The hub genes, which included SPI1, CD163, C1QB, SIGLEC1, C1QA, ITGAM, CD14, FCGR1A, VSIG4 and C1QC, were identified. From the interaction network analysis, 65 transcription factors (TFs) were found to be the regulatory signals. Some infections, inflammation and liver diseases were found to be most coordinated with the hub genes. Importantly, Paricalcitol, 3,3′,4,4′,5-Pentachlorobiphenyl, PD 98059, Medroxyprogesterone acetate, Dexamethasone and Tretinoin HL60 UP have shown possibility as therapeutic agents against COVID-19 and MUO.

**Conclusion:** This study provides new clues and references to treat both COVID-19 and MUO.

## 1 Introduction

Severe acute respiratory syndrome coronavirus 2 (SARS-COV-2) is a highly contagious coronavirus responsible for the life-threatening COVID-19. By December 2022, more than 650 million people were infected by SARS-COV-2, and it also caused more than 6 million deaths in more than 300 countries and regions around the world (WHO’s Monthly Operational Update on Health Emergencies, 2022). The risk of severe symptoms and complications, mortality and hospitalization in COVID-19 patients have been recently reported to be significantly increased due to some unhealthy pre-existing conditions, including hypertension, type 2 diabetes, and obesity (Fang et al., 2020). Compared with metabolic healthy patients, patients with metabolic disturbances also were observed to have a much poorer prognosis (Hu and Wang, 2021). Additionally, it has been reported that obesity can severely hamper the immune cells responsiveness to weaken the long-term protection against SARS-COV-2 (Stefan et al., 2021). Metabolic disturbances are a common complication of obesity, and only 15%–20% of obese patients are not affected by them (Soverini et al., 2010). Metabolic disturbances have been proven to be a significant aggravating factor in many obesity-related diseases, so the underlying mechanisms linking COVID-19 and metabolic unhealthy obesity (MUO) are need to be better clarified.

Angiotensin Converting Enzyme 2 (ACE2), whose gene expression is found in many human tissues, has been known to play a vital role as a receptor for SARS-CoV-2 entry and infection in target cells (Yan et al., 2020). MUO is a systemic disease, which has been discovered to be associated with the upregulated ACE2 expression in tissues in the whole human body (Zheng et al., 2020). As reviewed by Goossens et al., with the increased number of adipose tissues, the ACE2 expression is upregulated, making the concentration of SARS-COV-2 in adipocytes significantly higher than in other tissue cells, becoming a viral reservoir for SARS-CoV-2 (Goossens et al., 2020). Additionally, the contribution of MUO-related upregulated Transmembrane Serine Protease 2 (TMPRSS2) expression, hyperglycemia, and weakened immune surveillance to the poorer prognosis of COVID-19 in the corresponding patient population are also being in-depth investigated. This serves to demonstrate the potential importance of interaction between MUO and COVID-19.

**FIGURE 1:**
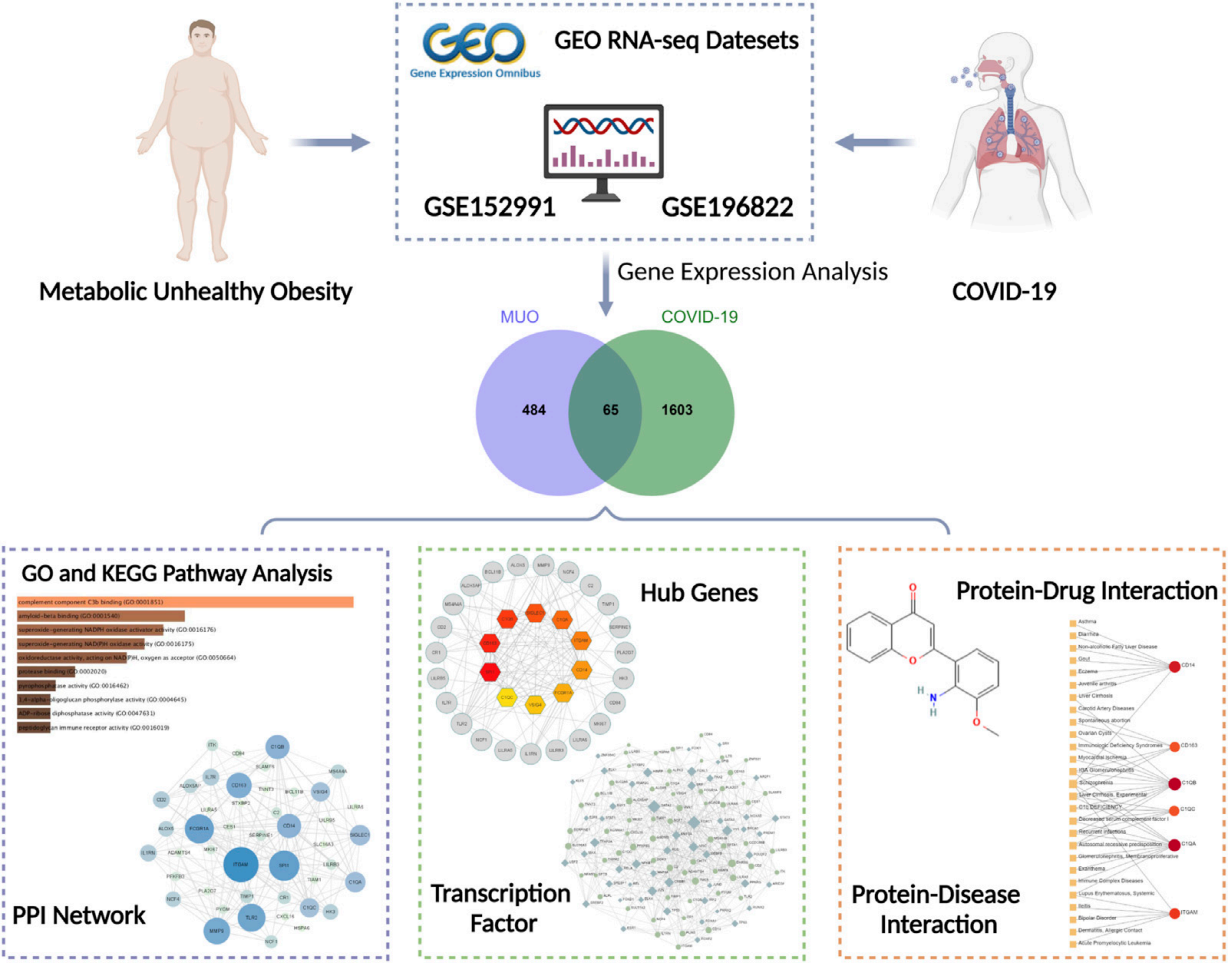
Overall flowchart of this research. Several analyses were conducted based on the gene expression analysis of datasets (GSE152991 and GSE196822) acquired from the GEO database.

In this study, we compared transcriptional profiles of COVID-19 with MUO patients. Two datasets were collected from the Gene Expression Omnibus (GEO) database, where GSE196822 (Banerjee et al., 2022) for COVID-19 and GSE152991 (Cifarelli et al., 2020) for MUO. Shared Differentially Expressed Genes (DEGs) were identified through cross-analysis of the two datasets, and the result was used to discover the relevant signaling pathways as well as the genes that are potential therapeutic targets for patients with MUO and COVID-19. Also, efforts to reveal the molecular mechanisms linking MUO and COVID-19 were made, including predictive transcription factor -gene interaction, protein-drug interaction, and gene-disease network analysis based on the identified DEGs. We further determined the association with other diseases that might provide further insights for the study about complications of and treatments for COVID-19. Figure 1 demonstrates the workflow of the entire system biology and bioinformatics analysis. We hope that the findings of this study will provide preliminary information that may help to understand the interaction between COVID-19 and MUO and help in selecting proper drugs and inventing future treatments that can combat COVID-19 and MUO.

## 2 Methods

### 2.1 Gene expression datasets

In this study, the RNA-seq data were obtained from the GEO database (https://www.ncbi.nlm.nih. gov/geo/) of the National Center for Biotechnology Information (NCBI). The GEO accession ID of the COVID-19 dataset is GSE196822, which is transcriptional profiling of COVID-19 whole blood samples through high throughput sequencing Illumina HiSeq 4,000 (*Homo sapiens*) for RNA sequence extraction. The COVID-19 dataset contains thirty-four infection samples and nine healthy controls (Supplementary Table S1). Besides, the GSE152991 dataset consists of twenty metabolic unhealthy obese subcutaneous adipose samples and eleven healthy controls. The adipose samples were sequenced by a high-throughput sequencing system called Illumina NovaSeq 6,000 (*Homo sapiens*).

### 2.2 Identification of DEGs and common DEGs among COVID-19 and MUO

A gene is characterized as DEG when tested at the transcriptional level under different conditions, and there were significant differences. The purpose of this analysis is to obtain DEGs for the datasets. The DEGs from GSE196822 were identified using DESeq2 (Love et al., 2014) of R programming language. Cutoff criteria (False Discovery Rate (FDR) < 0.05 and |log_2_ Fold Change| ≥ 1) was applied to obtain significant DEGs from both datasets. Intersection analysis was performed using an online analysis tool called Jvenn (http://bioinfo.genotoul.fr/jvenn.) to acquire the common DEGs of GSE196822 and GSE152991.

### 2.3 Gene ontology and pathway enrichment analysis

EnrichR (https://maayanlab.cloud/Enrichr/) was utilized to conduct gene ontology (biological process, cellular component and molecular function) and pathway enrichment analysis of common DEGs. Pathway enrichment analysis concluding Kyoto Encyclopedia of Genes and Genomes (KEGG), WikiPathways, Reactome and BioCarta were performed to identify the overlapped pathways among MUO and COVID-19. The adjusted *p*-value <0.05 was taken as a metric for quantifying the top-listed pathways and gene ontological processes.

### 2.4 Protein–protein interactions analysis

The common DEGs were used to construct protein subnetworks and further reveal the protein interactions between MUO and COVID-19. STRING—a protein interactome database is used in this analysis. The protein–protein interaction (PPI) network was constructed and visually represented by Cytoscape (v3.9.1).

### 2.5 Hub gene extraction

Nodes, edges, and their connections establish the PPI network. The nodes are the essential component of the network, and the most involved ones are regarded as the hub genes. Cytohubba (http://apps.cytoscape.org/apps/cytohubba) is a novel Cytoscape—plugin for ranking and extracting potential or targeted elements of a biological network based on various network features. The top 10 genes were identified depending on the degree algorithm, a commonly used centrality criterion. Among the 11 methods provided by Cytohubba that can be used to investigate networks from viewpoints, Maximal Clique Centrality (MCC) (Chin et al., 2014) has the best accuracy and effectiveness, which is used to identify the top 10 hub genes from the PPI network.

### 2.6 Recognition of transcription factors engaged with common DEGs

The transcription rate of a gene is closely related to the regulation of the TFs that attach to it. Therefore, TFs are very important for exploring gene activity at the molecular level. The topologically credible TFs are located using the NetworkAnalyst platform (3.0) (http://www.networkanalyst.ca) from the JASPAR database (http://jaspar.genereg.net/).

### 2.7 Suggested drug analysis

In this analysis, the protein–drug interactions have been predicted using the hub genes that COVID-19 shared with MUO based on the DSigDB database via Enricher. DSigDB database is a new gene set resource that related drugs/compounds and their target genes for gene set enrichment analysis (Yoo et al., 2015). DrugBank (www.drugbank.ca) Database and Comparative Toxicogenomics Database (http://ctdbase.org/) are used to screen out the drugs that have not been approved by the Food and Drug Administration (FDA).

### 2.8 Gene–disease association analysis

DisGeNET is a comprehensive database of gene–disease associations that synchronizes the biomedical characteristics of many diseases to determine the relationship between genes and specific diseases. It emphasizes the critical role of key genes in the occurrence and development of diseases. The gene-disease relationship via NetworkAnalyst was examined to reveal correlated diseases and their complications with hub genes based on the DiGeNET database.

## 3 Results

### 3.1 Identification of DEGs and common DEGs among MUO and COVID-19

**FIGURE 2:**
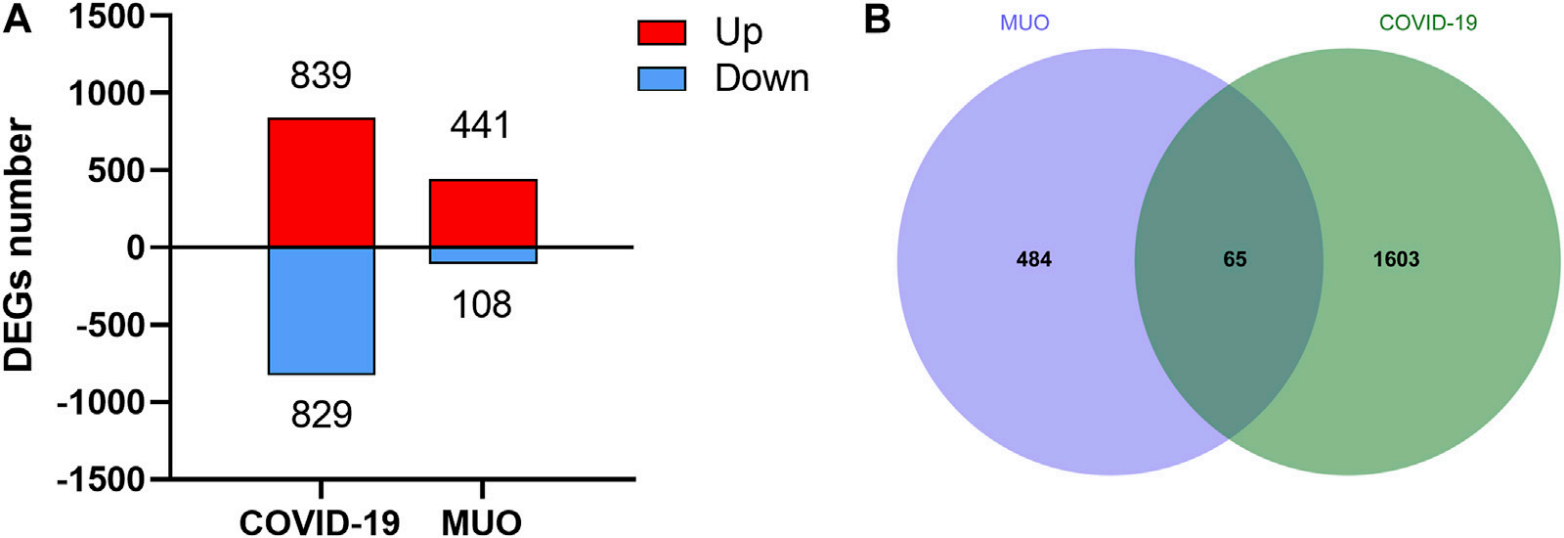
The differential expression levels of COVID-19 and MUO. **(A)** Number of DEGs at transcriptional levels. The red and blue bars refer to the number of upregulated and downregulated genes, respectively. **(B)** Venn diagram showing common DEGs of MUO and COVID-19.

To study the relationship and interaction between MUO and COVID-19, an analysis of the human transcriptomic datasets from the GEO database was conducted to identify and classify the common differentially expressed genes of MUO and COVID-19. The expression abundance information of transcriptome was shown in Supplementary Table S2 and Supplementary Table S3. Firstly, 1,668 DEGs were identified for COVID-19, including 839 upregulated and 829 downregulated DEGs (Figure 2A and Supplementary Table S4). In the same way, 441 upregulated and 108 downregulated DEGs for MUO were selected after completing the different statistical analysis processes (Figure 2A and Supplementary Table S5). Secondly, 65 common DEGs from MUO and COVID-19 datasets were identified after performing the intersection analysis (Figure 2B). These results suggested there are some similarities between MUO and COVID-19.

### 3.2 Gene ontology and pathway enrichment analysis

**FIGURE 3:**
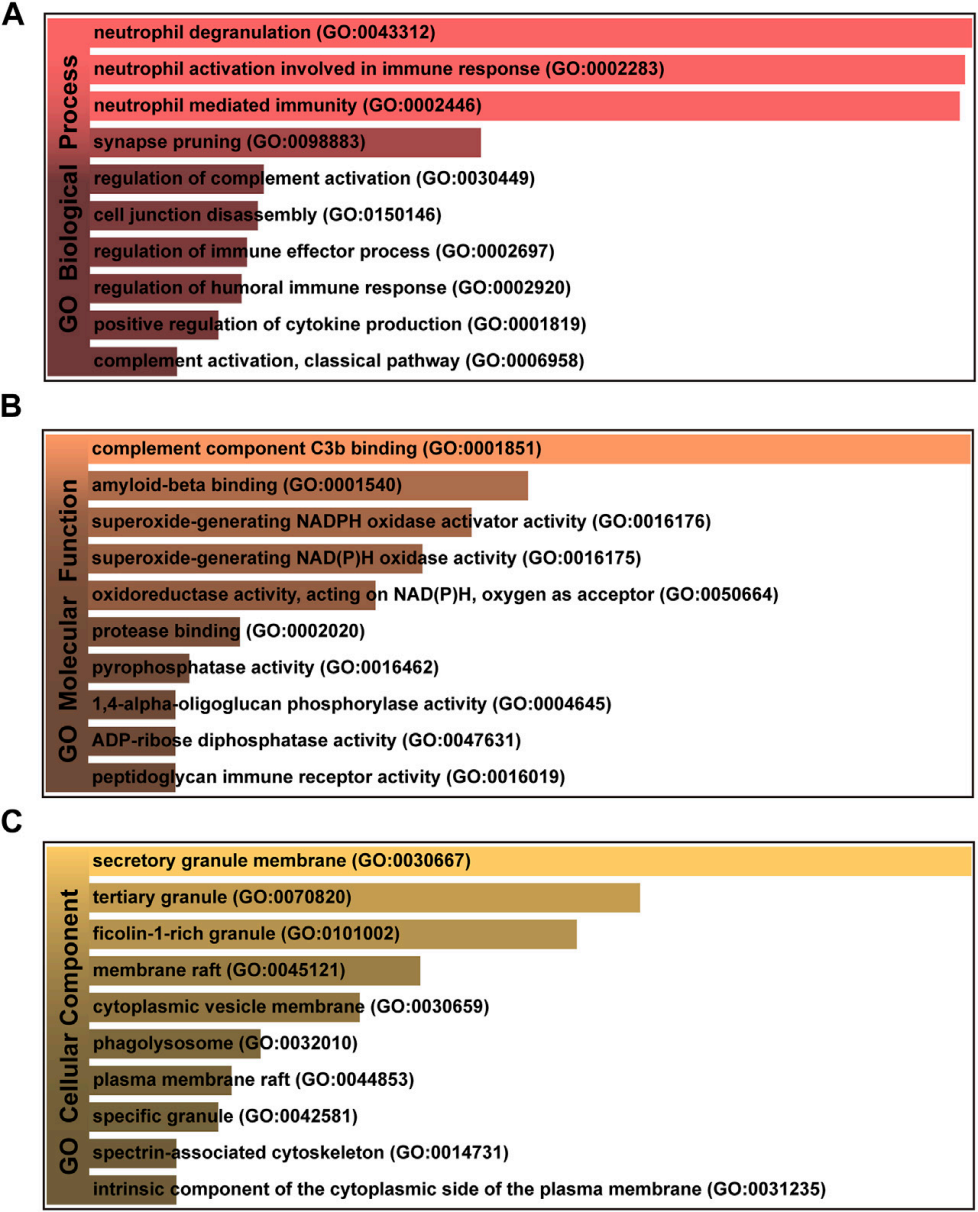
The bar graphs of gene ontology enrichment analysis of shared DEGs among MUO and COVID-19. **(A)** Biological Process, **(B)** Molecular Function, and **(C)** Cellular Component. Bar chart color depth represents significance. The lighter the color, the more significant it is.

To further investigate the functions and components of common DEGs, the gene ontology analysis has been performed. The top 10 terms in the biological process, molecular function and cellular component categories are summarized in Table 1. Figure 3 shows that the most significantly ontology terms including neutrophil-related terms, complement component C3b binding and granule-related terms.

**TABLE 1:** Ontological analysis of common DEGs between MUO and COVID-19.

Category	GO ID	Term	Adjusted *p*-values	Genes
GO Biological Process	GO:0043312	neutrophil degranulation	7.43E-10	ITGAM/CR1/NFAM1/STXBP2/HSPA6/SLC2A5/LILRB3/MMP9/CHIT1/HK3/TRPM2/DOK3/VNN1/ALOX5/CD14/TLR2
	GO:0002283	neutrophil activation involved in immune response	7.43E-10	ITGAM/CR1/NFAM1/STXBP2/HSPA6/SLC2A5/LILRB3/MMP9/CHIT1/HK3/TRPM2/DOK3/VNN1/ALOX5/CD14/TLR2
	GO:0002446	neutrophil mediated immunity	7.43E-10	ITGAM/CR1/NFAM1/STXBP2/HSPA6/SLC2A5/LILRB3/MMP9/CHIT1/HK3/TRPM2/DOK3/VNN1/ALOX5/CD14/TLR2
	GO:0098883	synapse pruning	2.64E-06	C1QB/C1QA/ITGAM/C1QC
	GO:0030449	regulation of complement activation	9.05E-05	C1QB/C1QA/CR1/C2/C1QC
	GO:0150146	cell junction disassembly	9.05E-05	C1QB/C1QA/C1QC
	GO:0002697	regulation of immune effector process	9.05E-05	C1QB/C1QA/CR1/C2/C1QC
	GO:0002920	regulation of humoral immune response	9.05E-05	C1QB/C1QA/CR1/C2/C1QC
	GO:0001819	positive regulation of cytokine production	1.21E-04	CD2/CCDC88B/ITK/NFAM1/SERPINE1/SLAMF6/CD14/TLR2/LILRA5
	GO:0006958	complement activation, classical pathway	2.27E-04	C1QB/C1QA/C1QC
GO Molecular Function	GO:0001851	complement component C3b binding	4.47E-05	CR1/ITGAM/VSIG4
	GO:0001540	amyloid-beta binding	9.14E-03	C1QA/ITGAM/LILRB3/TLR2
	GO:0016176	superoxide-generating NADPH oxidase activator activity	1.31E-02	NCF1/NCF4
	GO:0016175	superoxide-generating NAD(P)H oxidase activity	1.92E-02	NCF1/NCF4
	GO:0050664	oxidoreductase activity, acting on NAD(P)H, oxygen as acceptor	2.91E-02	NCF1/NCF4
	GO:0002020	protease binding	9.65E-02	ADAMTS4/SERPINE1/TIMP1
	GO:0016462	pyrophosphatase activity	9.65E-02	TRPM2/ALPL
	GO:0004645	1,4-alpha-oligoglucan phosphorylase activity	9.65E-02	PYGM
	GO:0047631	ADP-ribose diphosphatase activity	9.65E-02	TRPM2
	GO:0016019	peptidoglycan immune receptor activity	9.65E-02	CD14
GO Cellular Component	GO:0030667	secretory granule membrane	1.46E-06	TRPM2/ITGAM/CR1/DOK3/VNN1/NFAM1/CD14/SLC2A5/LILRB3/TLR2
	GO:0070820	tertiary granule	4.09E-05	CHIT1/TRPM2/ITGAM/CR1/DOK3/STXBP2/MMP9
	GO:0101002	ficolin-1-rich granule	5.89E-05	HK3/TRPM2/CR1/DOK3/ALOX5/HSPA6/MMP9
	GO:0045121	membrane raft	2.97E-04	ITGAM/CR1/NFAM1/CD14/MS4A4A/TLR2
	GO:0030659	cytoplasmic vesicle membrane	4.95E-04	TRPM2/CD163/CR1/DOK3/NCF4/CD14/LILRB3/TLR2
	GO:0032010	phagolysosome	1.38E-03	NCF1/NCF4
	GO:0044853	plasma membrane raft	1.68E-03	ITGAM/CR1/KCNMA1/MS4A4A
	GO:0042581	specific granule	1.73E-03	CHIT1/TRPM2/ITGAM/STXBP2/SLC2A5
	GO:0014731	spectrin-associated cytoskeleton	2.30E-03	SPTA1/SPTB
	GO:0031235	intrinsic component of the cytoplasmic side of the plasma membrane	2.30E-03	SPTA1/SPTB

**FIGURE 4:**
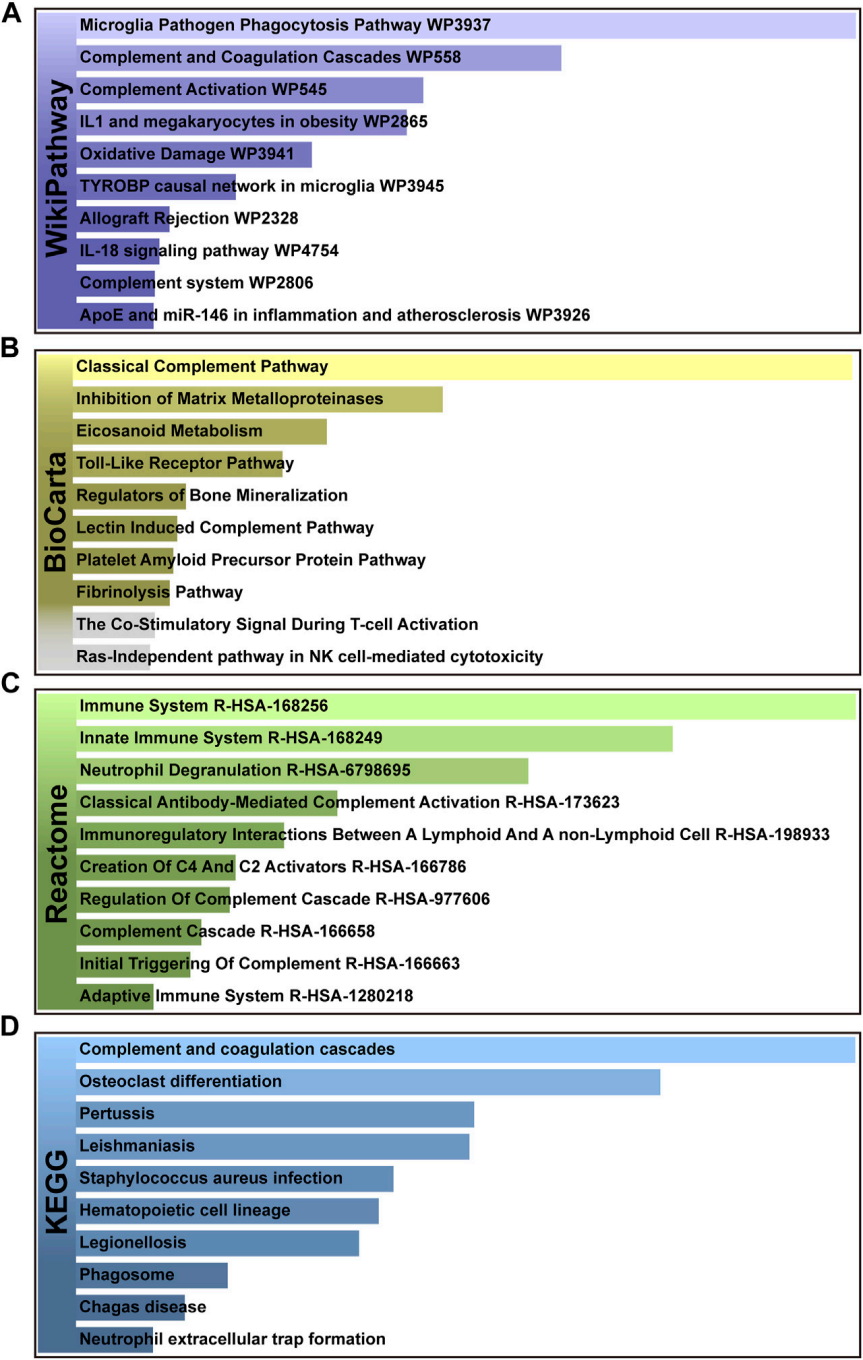
The bar graphs of pathway enrichment analysis of shared DEGs among MUO and COVID-19. **(A)** WikiPathways, **(B)** BioCarta, **(C)** Reactome, and **(D)** KEGG. Bar chart color depth represents significance. The lighter the color, the more significant it is.

In addition, pathways analysis was performed to reveal the interaction between MUO and COVID-19 through basic molecular or biological processes. WikiPathways, BioCarta, Reactome and KEGG databases were used for pathway analysis. The top 10 enriched pathways of the shared DEGs obtained from the selected database are enlisted in Table 2. For the result demonstrated in Figure 4, the top significant pathways were microglia pathogen phagocytosis (WikiPathways), inhibition of matrix metalloproteinases (BioCarta), immune system (Reactome), complement and coagulation cascades (KEGG) and osteoclast differentiation (KEGG).

**TABLE 2:** Pathway enrichment analysis of common DEGs between MUO and COVID-19.

Category	Pathways	Adjusted *p*-values	Genes
WikiPathways	Microglia Pathogen Phagocytosis Pathway	6.11E-09	C1QB/C1QA/ITGAM/NCF1/NCF4/FCGR1A/C1QC
	Complement and Coagulation Cascades	2.14E-06	C1QB/C1QA/CR1/SERPINE1/C2/C1QC
	Complement Activation	3.08E-05	C1QB/C1QA/C2/C1QC
	IL1 and megakaryocytes in obesity	3.34E-05	TIMP1/MMP9/PLA2G7/TLR2
	Oxidative Damage	2.21E-04	C1QB/C1QA/C2/C1QC
	TYROBP causal network in microglia	9.99E-04	CD84/ITGAM/CXCL16/C1QC
	Allograft Rejection	3.74E-03	C1QB/C1QA/C2/C1QC
	IL-18 signaling pathway	3.74E-03	NCF1/TIMP1/MMP9/ACACB/PLA2G7/CXCL16
	Complement system	3.74E-03	CR1/VSIG4/TLR2/C2
	ApoE and miR-146 in inflammation and atherosclerosis	3.74E-03	SPI1/TLR2
BioCarta	Classical Complement Pathway	1.35E-07	C1QB/C1QA/C2/C1QC
	Inhibition of Matrix Metalloproteinases	2.88E-04	TIMP1/MMP9
	Eicosanoid Metabolism	2.52E-03	ALOX5/ALOX5AP
	Toll-Like Receptor Pathway	5.77E-03	CD14/TLR2
	Regulators of Bone Mineralization	3.52E-02	ALPL
	Lectin Induced Complement Pathway	4.14E-02	C2
	Platelet Amyloid Precursor Protein Pathway	4.46E-02	SERPINE1
	Fibrinolysis Pathway	4.77E-02	SERPINE1
	The Co-Stimulatory Signal During T-cell Activation	6.31E-02	ITK
	Ras-Independent pathway in NK cell-mediated cytotoxicity	6.91E-02	CD2
Reactome	Immune System	6.39E-11	C1QB/LILRA6/ITK/C1QA/IL1RN/ITGAM/NCF1/NCF4/STXBP2/SLC2A5/LILRA5/TRPM2/ALOX5/SLAMF6/TIMP1/FCGR1A/CR1/BCL11B/NFAM1/HSPA6/MMP9/LILRB5/CHIT1/DOK3/VNN1/SIGLEC1/IL7R/TLR2/C1QC
	Innate Immune System	5.71E-09	C1QB/LILRA6/ITK/C1QA/ITGAM/CR1/NCF1/NFAM1/NCF4/HSPA6/SLC2A5/MMP9/CHIT1/TRPM2/DOK3/VNN1/ALOX5/FCGR1A/TLR2/C1QC
	Neutrophil Degranulation	2.27E-07	LILRA6/ITGAM/CR1/NFAM1/HSPA6/SLC2A5/MMP9/CHIT1/TRPM2/DOK3/VNN1/ALOX5/TLR2
	Classical Antibody-Mediated Complement Activation	3.82E-05	C1QB/C1QA/C1QC
	Immunoregulatory Interactions Between A Lymphoid And A non-Lymphoid Cell	1.38E-04	LILRA6/SLAMF6/SIGLEC1/FCGR1A/LILRB5/LILRA5
	Creation Of C4 And C2 Activators	4.55E-04	C1QB/C1QA/C1QC
	Regulation Of Complement Cascade	4.60E-04	C1QB/C1QA/CR1/C1QC
	Complement Cascade	8.99E-04	C1QB/C1QA/CR1/C1QC
	Initial Triggering of Complement	1.09E-03	C1QB/C1QA/C1QC
	Adaptive Immune System	2.79E-03	LILRA6/ITK/NCF1/NCF4/SLAMF6/SIGLEC1/FCGR1A/LILRB5/TLR2/LILRA5
KEGG	Complement and coagulation cascades	3.15E-10	C1QB/C1QA/ITGAM/CR1/SERPINE1/VSIG4/C2/C1QC
	Osteoclast differentiation	7.89E-09	LILRA6/SPI1/NCF1/NCF4/FCGR1A/LILRB3/LILRB5/LILRA5
	Pertussis	1.70E-07	C1QB/C1QA/ITGAM/CD14/C2/C1QC
	Leishmaniasis	1.84E-07	ITGAM/CR1/NCF1/NCF4/FCGR1A/TLR2
	*Staphylococcus aureus* infection	6.45E-07	C1QB/C1QA/ITGAM/FCGR1A/C2/C1QC
	Hematopoietic cell lineage	8.23E-07	CD2/ITGAM/CR1/CD14/FCGR1A/IL7R
	Legionellosis	1.14E-06	ITGAM/CR1/HSPA6/CD14/TLR2
	Phagosome	9.96E-06	ITGAM/NCF1/NCF4/CD14/FCGR1A/TLR2
	Chagas disease	2.03E-05	C1QB/C1QA/SERPINE1/TLR2/C1QC
	Neutrophil extracellular trap formation	3.42E-05	ITGAM/CR1/NCF1/NCF4/FCGR1A/TLR2

### 3.3 PPI network analysis and classification of hub genes

**FIGURE 5:**
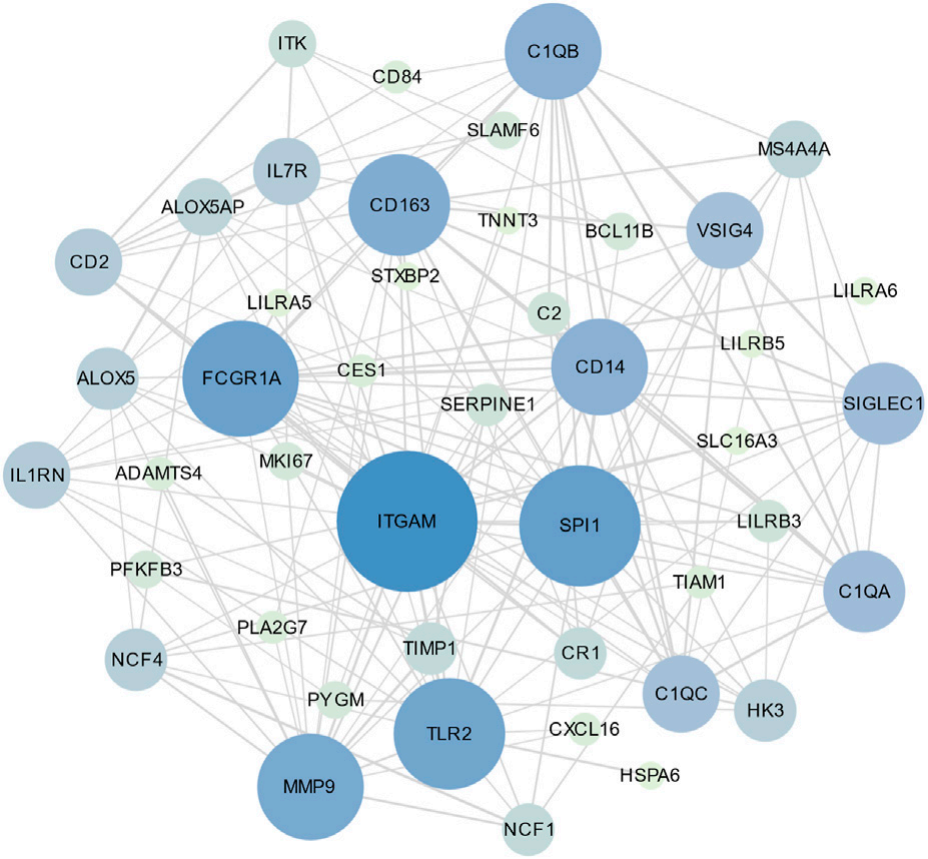
The PPI network of common DEGs between SARS-CoV-2 and MUO. The circle nodes represent the common DEGs and the edges represent the interaction between nodes. The PPI network has 65 nodes and 167 edges.

**FIGURE 6:**
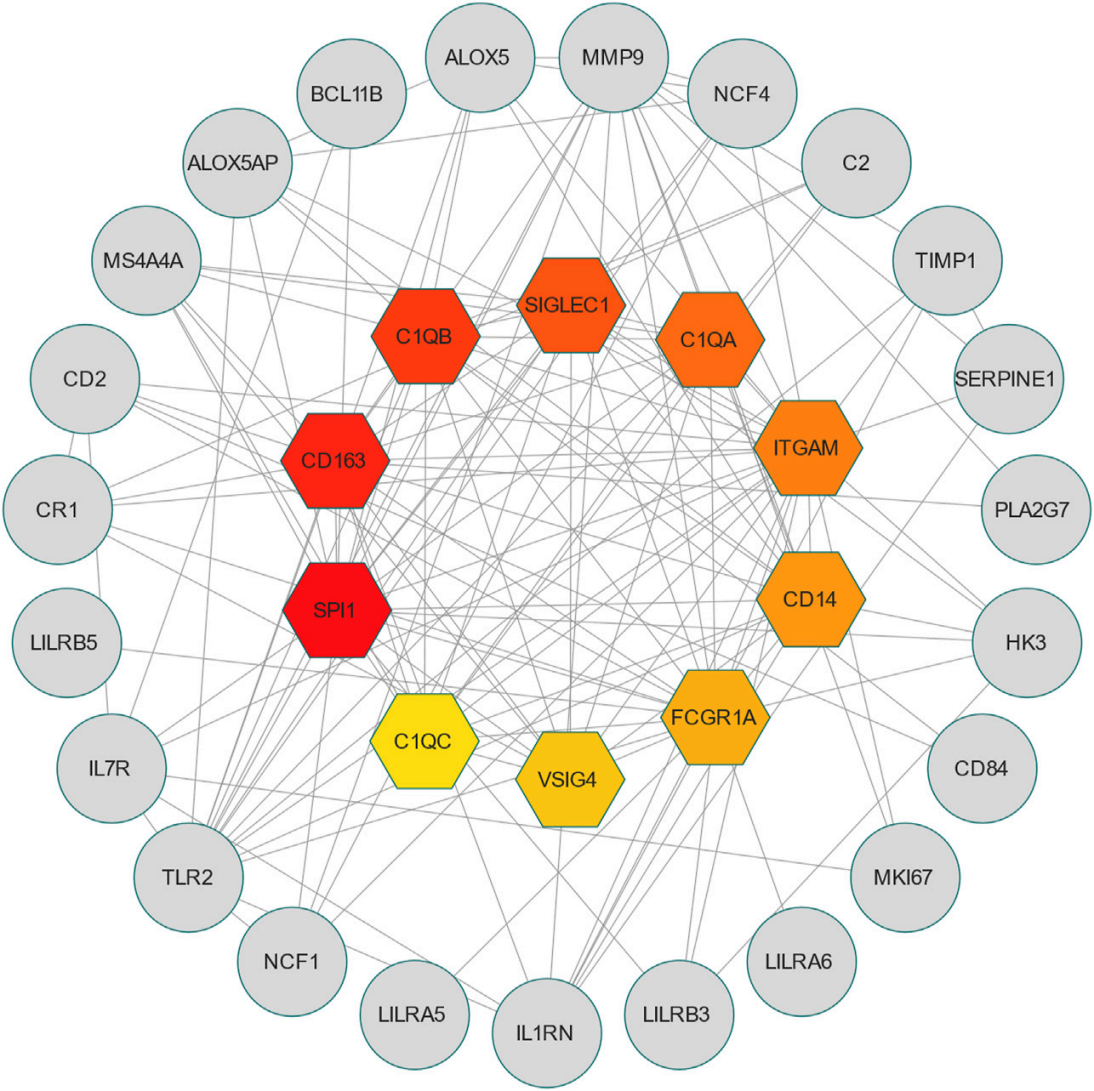
The hub genes were obtained from the PPI network. The colorful nodes indicate the highlighted top 10 hub genes and their interactions with other molecules. This network consists of 33 nodes and 141 edges.

PPI is an important part of the cellular biochemical response network and can be used to map the functional and structural knowledge of cellular protein networks (De Las Rivas and Fontanillo, 2010). Key proteins that affect how cells and systems function biologically have been discovered due to the assessment and analysis of the PPI networks (Song et al., 2023). To study the mechanisms of the interactions and connectivity between DEGs, the PPI network was constructed. This PPI network consists of 65 nodes and 167 edges and all the interconnected nodes are depicted in Figure 5. The most interconnected nodes in the PPI network are acknowledged as hub genes. The top 10 identified hub genes are, namely, SPI1, CD163, C1QB, SIGLEC1, C1QA, ITGAM, CD14, FCGR1A, VSIG4 and C1QC, which is depicted in Figure 6. Supplementary Table S6 shows the basic information of the hub genes.

### 3.4 Identification of TF–gene interactions for the common genes

**FIGURE 7:**
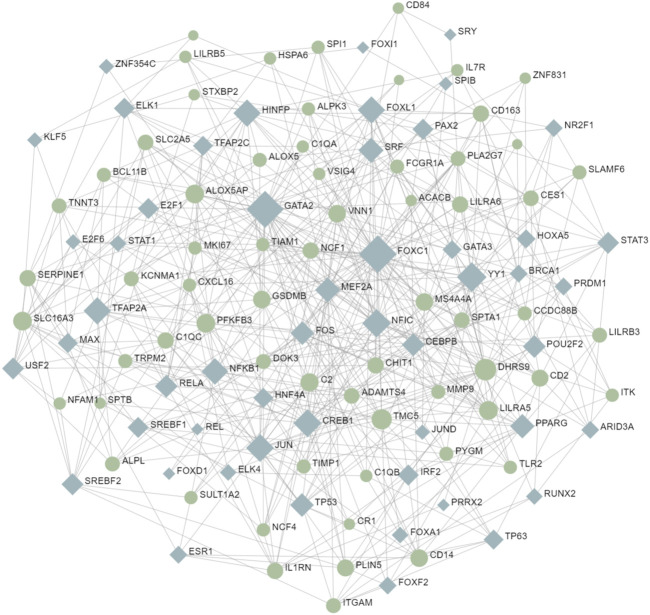
The interaction network between TFs and common DEGs. The circular and squared nodes represent the DEGs and the TFs, respectively.

Transcription factors are the molecules that control the activity of genes by determining whether the genes’ DNA is transcribed into RNA. Together, they make up a complex transcriptional regulatory network (Jiang et al., 2016). To understand the interaction between COVID-19 and MUO at the transcriptional level and further investigate the transcription factors regulatory network, the TFs were decoded through a network-based approach. The interaction network between common DEGs and TFs is depicted in Figure 7. From the interaction network analysis, 65 TFs were found to be the regulatory signals that regulate common DEGs, implying that they interact. Supplementary Table S7 shows the basic information of the top 10 transcription factors.

### 3.5 Identification of candidate drugs

To uncover the drug molecules for the therapy of MUO and COVID-19, the DSigDB database was used to extract the possible drug molecules based on the transcriptome signature. The top 6 potential effective drugs are screened based on their *p*-value of common DEGs. These drugs are Paricalcitol, 3,3′,4,4′,5-Pentachlorobiphenyl, PD 98059, Medroxyprogesterone acetate, Dexamethasone, Tretinoin HL60 UP. Table 3 shows detailed information concerning these drug compounds. The functions of these drugs respectively are vitamin D analog, inhibiting or antagonizing the action or biosynthesis of estrogenic compounds, inhibiting MAP-kinase kinase activation, a progestin analogue, a glucocorticoid, a vitamin A derivative. Among these drugs, in the vast majority of them, their function is related to the inhibition of inflammatory response. Given the fact that MUO patients have poorer immunity surveillance and higher inflammatory receptor expression the inflammation suppression is highlighted as the most promising target for selecting and developing drugs.

**TABLE 3:** The recommended drugs for COVID-19 and MUO.

Name	*p*-value	Chemical formula	Structure
Paricalcitol CTD 00003033	3.30E-08	C_27_H_44_O_3_	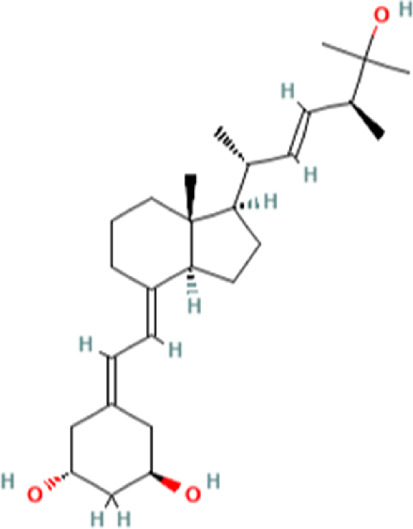
3,3′,4,4′,5-Pentachlorobiphenyl CTD 00001077	7.37E-08	C_12_H_5_Cl_5_	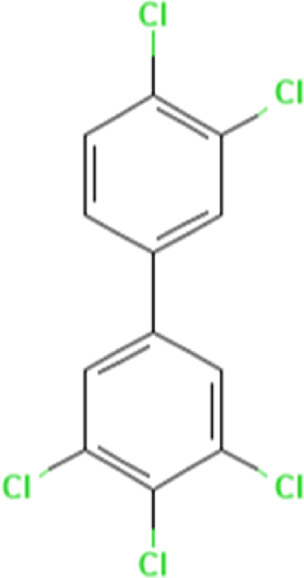
PD 98059 CTD 00003206	1.72E-07	C_16_H_13_NO_3_	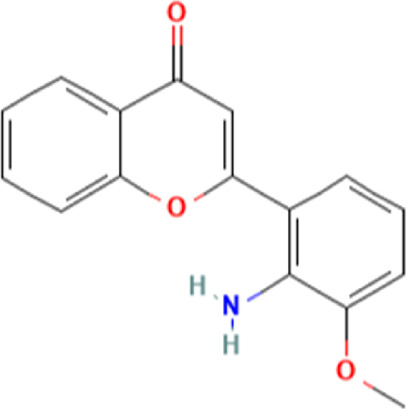
Medroxyprogesterone acetate CTD 00006623	4.53E-06	C_24_H_34_O_4_	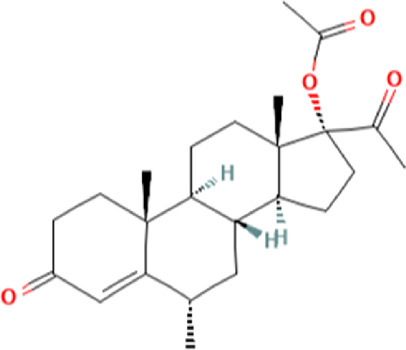
Dexamethasone CTD 00005779	8.56E-06	C_22_H_29_FO_5_	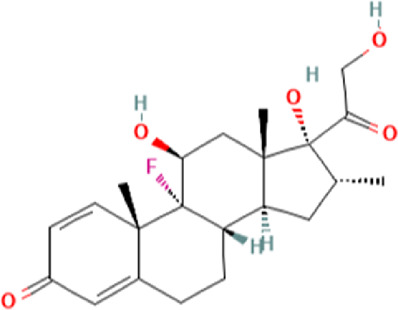
Tretinoin HL60 UP	1.30E-05	C_20_H_28_O_2_	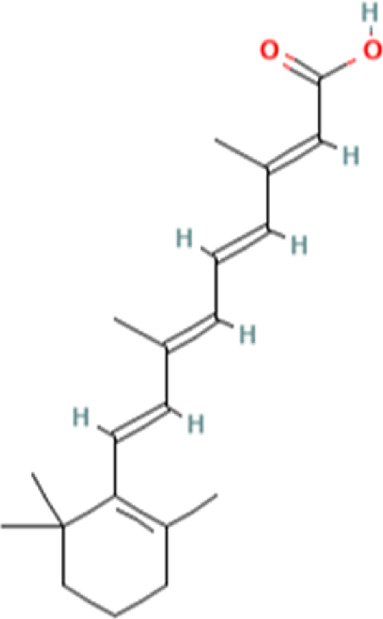

### 3.6 Identification of gene-disease associations

**FIGURE 8:**
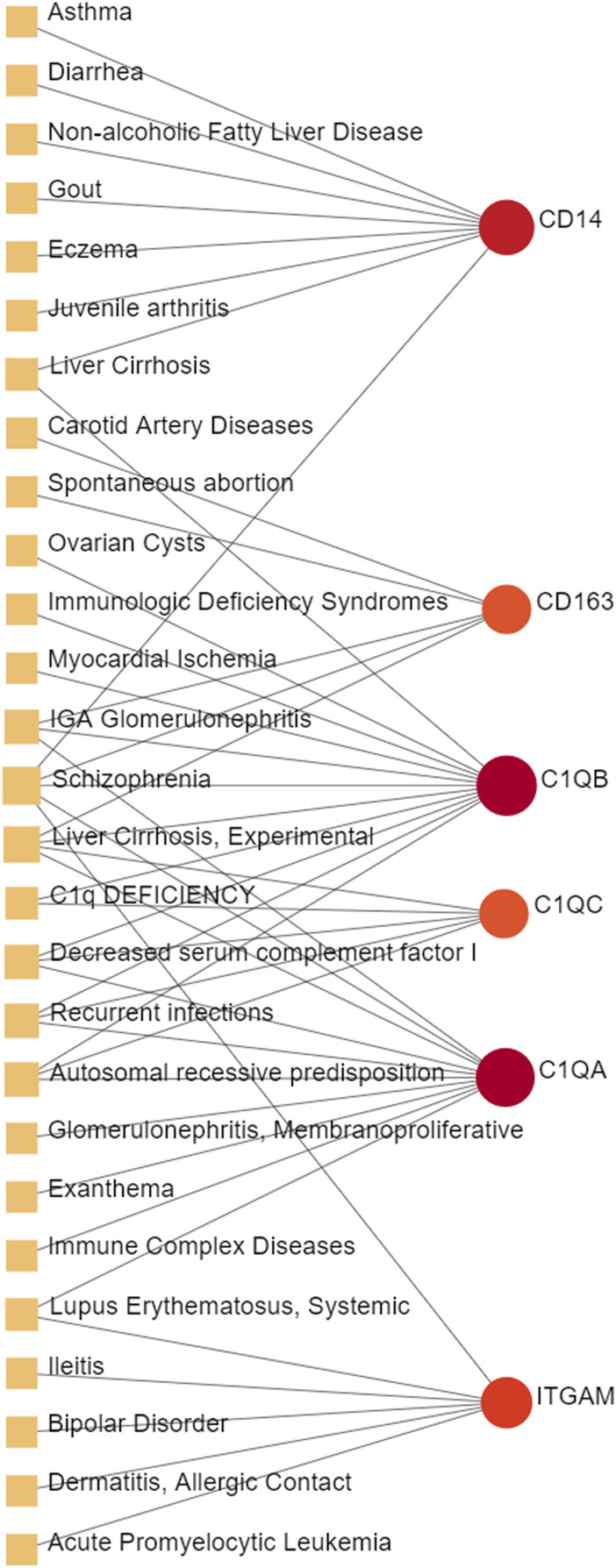
The gene-disease relationship network represents diseases associated with hub genes. Herein, the diseases characterized by the orange square and the red circle nodes indicate the gene symbols that interact with the disease. The color depth of the circle nodes depends on the number of diseases associated with the gene. The more diseases associated with this gene, the darker the node will be.

Different diseases can be associated are that they have one or more overlapped disease-associated genes (Barabási et al., 2011). Unveiling the correlation between different diseases is helpful to expand the indications of existing drugs so as to reduce medical costs from the perspective of health economics. In order to further identify the diseases most related to MUO and COVID-19, the gene-diseases relationship analysis was conducted to review the disease that were most coordinated with our reported hub genes, which is depicted in Figure 8. It showed that the Asthma, Non-alcoholic Fatty Liver Disease, Liver Cirrhosis, Recurrent infections and Glomerulonephritis were most coordinated with the hub genes identified in this study. Some of these identified diseases involve inflammation.

## 4 Discussion

MUO is a type of metabolic disorder regarded as the main obesity-related complication. Patients with MUO usually preserve insulin resistance, hypertension, chronic inflammation, and altered liver inflammation, all of which are negative preconditions for the prognosis of COVID-19 infection (Cifarelli et al., 2020). Here, MUO and COVID-19 transcriptomics analysis revealed that 65 shared DEGs show similar expression patterns in these two disorders.

GO pathway analysis was conducted to assess the biological importance of the identified common DEGs in the pathogenesis of MUO and COVID-19. Three types of GO analysis were conducted. Neutrophil-related pathways are among the top GO terms for the biological process, including neutrophil degranulation (GO:0043312), neutrophil activation involved in immune response (GO:0002446) and neutrophil mediated immunity (GO:0002283). Increasing evidence suggests that inhibiting neutrophil degranulation is beneficial for ameliorating inflammation-induced myocyte damage, hepatic acute phase response and thrombosis formation in severe COVID-19 patients (Gutmann et al., 2022). In the molecular function, complement component C3b binding (GO:0001851) and amyloid-beta binding (GO:0001540) are two top GO pathways. Concerning COVID-19, C3b recruits immune cells to the sites of infection and induces activation and further differentiation towards an inflammatory phenotype with the subsequent activation of lectin pathway (LP)-mediated C3b deposition, which is critical for the induction and maintenance of a severe inflammatory response to SARS-CoV-2 (Ali et al., 2021). Moreover, a previous study found that the raised level of C3b in serum is linked to obesity (Oberbach et al., 2011), which may provide essential insight into the severe inflammation that is occurred in patients with both MUO and COVID-19. In addition, recent research has proved that SARS-CoV-2 infection elevates neuroinflammation by inducing dysregulation of microglia and astrocyte subpopulations, alters brain structure leads to abnormal accumulation of amyloid-beta (Douaud et al., 2022). Similarly, the pathway of activating and modifying proinflammatory cells in the central neuron system (CNS) is also the way that MUO increases the risk of Alzheimer’s disease (Guo et al., 2021). It implicated the underlying synergistic effect of increasing Alzheimer’s disease occurrence on COVID-19 patients with MUO. The top GO terms based on the cellular component were secretory granule membrane (GO:0030667) and tertiary granule (GO:0070820). Besides the neutrophil degranulation, which has been discussed above, the secretory granule is essential for SARS-COV-2 exiting the cell. Intracellular transport of SARS-COV-2 within the cells involves secretory granules, each containing a single virus particle. These vesicles then fuse with the plasma membrane of host cells, releasing the virus outside the cell and facilitating the transmission of SARS-COV-2 (Eymieux et al., 2021). Yeming Yang et al., revealed that metabolic unhealthy disorders could alter the membrane phospholipid distribution in multiple physiological systems (Yang et al., 2021), affecting secretory granule membrane synthesis.

The top pathways terms from the four selected databases were complement and coagulation cascades (KEGG), osteoclast differentiation (KEGG), microglia pathogen phagocytosis (WikiPathways) and inhibition of Matrix Metalloproteinases (MM) (BioCarta). Here, complement and coagulation cascade activation are the common pathways in virus infection and metabolic disorders. COVID-19 induces the pro-inflammatory state, which stimulates uncontrolled activation of the complement system and neutrophil extracellular traps (NETs)-formation, both of which promote the coagulation cascade and induce a state of “thrombo-inflammation” (Borczuk and Yantiss, 2022). In patients with MUO, the dysregulation of secreted proteins and the secretory machinery in the liver lead to the abnormally regulated complement and coagulation cascades (Stocks et al., 2022), suggesting that this cascade can be an important target for developing alternative management measures for patients with both MUO and COVID-19. In addition, recent studies about patients diagnosed with COVID-19 have reported bone loss. Preliminary research showed that it might be due to the SARS-COV-2-induced cytokine dysregulation, as the circulating pro-inflammatory cytokines upregulate osteoclast differentiation in bone tissues (Qiao et al., 2022). Based on that, in MUO patients, the lipid-altering conditions can be aggravated by the altered activity and differentiation of osteoclast, which directly lead to more severe bone loss (Kim et al., 2021). Aside from the skeletal system lesions, the degenerative changes in CNS caused by the neuroinflammation has been identified in SARS-CoV-2 infection patients. It has been believed by some researchers that its underlying pathological pathways can be attributed to microglia pathogen phagocytosis, which is also the key in the sortilin-mediated microglia phagocytes production in obesity patients (Talbot et al., 2018; Ma et al., 2022). After COVID-19 patients were discharged from the hospitals, the pulmonary long-term consequences have been found to depend mainly on the activity of matrix metalloproteinases. The deregulated abnormal accumulation of extracellular matrix protein is crucial in the presence of LONG-COVID symptoms, since that the enzymes are deeply related to tissue repairment (Blanco et al., 2021). As for the obesity patients, the inhibition of matrix metalloproteinases has been proved to suppress glomerular inflammation and fibrogenesis (Niu et al., 2016).

Based on the identified DEGs, a PPI network was constructed to analyze interconnected proteins’ functional characteristics in-depth and predict potential drug targets. The hub genes essential in the pathogenesis of COVID-19 and MUO could be key drug targets and biomarkers in them. The top 10 hub genes associated with COVID-19 and MUO were retrieved through MCC method, including SPI1, CD163, C1QB, SIGLEC1, C1QA, ITGAM, CD14, FCGR1A, VSIG4, C1QC. Protein SPI1 is the essential transcription factor used to predict dysregulated hematopoiesis in bone marrow in severe COVID-19 patients. Recent evidence has proved the link between hematopoietic dysfunction of bone marrow and COVID-19, which is characterized by the accumulation of immature myeloid progenitors and a dramatic reduction of lymphoid progenitors (Wang et al., 2021). Obesity-related abnormal glucose metabolism has also been reported to impair bone marrow function, especially hematopoiesis (Liu et al., 2018). Therefore, patients with MUO precondition are more likely to suffer from severe SARS-COV-2 infection complication. Furthermore, the hub genes C1QA, C1QB, C1QC, VSIG4 and CD14 have been regarded as the main biomarkers for predicting macrophage and neutrophil mediated inflammation, especially in the respiratory and cardiovascular systems (Zhang and Zhang, 2022). They are also the essential biomarkers that have been identified in the unfavorable metabolic profile of MUO patients (Chang et al., 2019). Additionally, during the recovery process of patients, especially in the early recovery stage (ERS), the ratio of classical CD14^+^ monocytes was reported to be elevated, accompanied by high expression of inflammatory genes, which prevented patients from achieving a better prognosis. The hub protein ITGAM is an essential molecular receptor that plays a vital role in many complements mediated pathways that aggravate the COVID-19 symptoms, such as the C5a-C5aR1 axis in the pathophysiology of acute respiratory distress syndrome (Carvelli et al., 2020). It is also a critical marker for inflammatory macrophages, whose infiltration can be elevated due to obesity (Moreno-Indias et al., 2016). Bronchoalveolar lavage fluid CD4^+^ T cells of patients with COVID-19 were T_H_1-skewed and showed de-repression of genes downregulated by Vitamin D which notably activates the recruiting of c-JUN and switches on the pro-inflammatory programs of T_H_1 cells (Chauss et al., 2022). This c-JUN mediated cascade has also been discovered to be activated by increased metabolic stress caused by obesity (Wang et al., 2020). Based on this information, we can speculate that the induced raised degree of macrophage infiltration and transcriptional factors activation by glucose metabolism and oxidative phosphorylation can lead to a poorer prognosis and higher mortality of COVID-19.

TFs mainly regulated transcription and expression of target genes. This result revealed transcription factors regulator network in the COVID-19 and MUO, including FOXC1, GATA2, YY1, DHRS9, NFIC, CREB1, JUN, TMC5, HINFP, and FOXL1. In previous bioinformatics analysis, several separate studies revealed that some essential TFs, including FOXC1, GATA2, YY1 and FOXL1, are associated with SARS-CoV-2 infection (Islam et al., 2020; Auwul et al., 2021; Ahmed et al., 2022; Lu et al., 2022; Hossain et al., 2023). GATA2 is associated with hematopoietic and immune deficiency of COVID-19 (Collin et al., 2015). JUN is a subunit of activating protein 1, an inducible transcription factor composed of multiple protein complexes, that plays a role in several types of cell differentiation and inflammation (Chang et al., 2013). Moreover, CASP1 is the most involved pathway in cytokine storm, and YY1 is a crucial TF in CASP1 expression (Zheng et al., 2022). In obese mouse models, YY1 was markedly upregulated (Lu et al., 2014), which may provide us with the possible cause of increased mortality in COVID-19 patients with MUO.

Several drugs have been extracted as candidate treatments against COVID-19 and MUO. We discovered 3,3′,4,4′,5-Pentachlorobiphenyl, a compound that inhibits or antagonizes the action or biosynthesis of estrogenic compounds, as the candidate drug for treating COVID-19. This may reason for the observed moderate decrease of pulmonary ACE2 expression after administration of the androgen receptor antagonist enzalutamide, which is documented in recent studies (Lott et al., 2022). This effect may play a positive role in reducing the incidence of respiratory complications in patients with MUO. Another extracted drug was PD 98059, which is applied for the inhibition of MAP-kinase (MAPK) kinase activation. The MAPK pathway is one of the most involved pathways causing cytokine storm in patients with severe COVID-19 (COvid-19 Multi-omics Blood ATlas COMBAT ConsortiumCOvid-19 Multi-omics Blood ATlas COMBAT Consortium, 2022; Geng et al., 2021). The inhibition of this pathway is a promising therapeutic target for preventing severe COVID-19. PD 98059 can relieve obesity induced by high fat diet (Zheng et al., 2023). Moreover, this study found medroxyprogesterone acetate, dexamethasone and tretinoin as potential drugs. Medroxyprogesterone acetate, a progestin, was recently tested as a drug for HIV patients (Vanpouille et al., 2021). Several clinical trials have been carried out to study dexamethasone’s therapeutic effect on COVID-19 infection. Many results have proved that it can regulate inflammation-mediated lung injury (Horby et al., 2021), thereby reducing the progress of respiratory failure and death; at the same time, attention has been paid to its positive effect on neutrophil aggregation and interferon system imbalance caused by COVID-19 (Sinha et al., 2022). Moreover, as has been shown by drug tests in human cell lines and human lower respiratory organoids, tretinoin, as a derivative of vitamin A, has antiviral activity against all SARS-COV-2 antibodies (Tong et al., 2022). Several studies have indicated that carotenoids and carotenoid conversion products with provitamin A activity have anti-obesity activity (Gomes et al., 2021).

The gene-disease analysis was performed to predict relationships between common DEGs and different disorders. The analysis showed various diseases correlated with MUO and COVID-19, including liver, immune and cardiovascular system disorders. Liver cirrhosis has also been found in our gene-disease network; the patients with it have a higher probability of severe COVID-19 symptom, particularly high rates of hepatic decompensation and death (Marjot et al., 2021). Furthermore, COVID-19 infection and MUO have been shown to cause and aggravate various chronic liver diseases (Steenblock et al., 2021; Kouvari et al., 2022). The immune system diseases also showed a strong correlation in the gene-disease network. IGA Glomerulonephritis is a kind of immunoglobulin A related inflammation and usually occurs following viral infection. Increasing evidence supports that COVID-19 infection and vaccination against SARS-CoV-2 may trigger or exacerbate IGA Glomerulonephritis (Bronz et al., 2022). Another strongly correlated immune disease identified in the network is Lupus Erythematosus, Systemic. During the COVID-19 pandemic, patients with Lupus Erythematosus, Systemic have lowered risk of COVID-19, hospitalized COVID-19 or severe COVID-19 (Ran et al., 2022), suggesting that this specific precondition blocks the entry of the virus and transmission in some way. Moreover, the involvement of myocardial ischemia in COVID-19 is directly associated with cytokine-mediated plaque destabilization and hypercoagulability, which induce ischemic stroke and acute myocardial infarction (Modin et al., 2020). Additionally, there is a significant correlation between metabolic disorders and prolonged QTc interval, which is the early marker of transient myocardial ischemia (Guo et al., 2017).

In addition to presenting some interesting findings, our study has some limitations. These results, including DEGs and candidate drug identification, as well as all the network analysis, were obtained by bioinformatics and system biology approach. The results need further experimental verification. Additionally, the selected datasets include different groups of people with two different diseases, rather than the same population with both MUO and COVID-19. Thus, further studies and clinical trials are needed to validate the biological functions of the identified hub genes and the safety and efficacy of the specified candidate drugs, as well as their pharmacological characteristics.

## Data Availability

The datasets presented in this study can be found in online repositories. The names of the repository/repositories and accession number(s) can be found in the article/Supplementary Material.
