# Supplementary material for: Bioinformatics and system biology approach to identify the influences of SARS-CoV-2 on metabolic unhealthy obese patients
Source: Front Mol Biosci. 2023 Oct 9;10:1274463. doi: 10.3389/fmolb.2023.1274463 (PMC10591333; doi:10.3389/fmolb.2023.1274463)
Supplement: Supplementary file 3 [file Table1.DOCX]

Supplementary Table 1. The baseline characteristic of sample in GSE196822.

| Category | Healthy Control (n=9) | Asymptomatic (n=8) | Mild (n=9) | Moderate (n=10) | Severe (n=7) |
| --- | --- | --- | --- | --- | --- |
| Age, years | 31.4±13.6 | 35.4±25.6 | 50.7±34.3 | 52.7±17.3 | 62.1±17.9 |
| Sex |  |  |  |  |  |
| Male | 5 (55.6%) | 1 (12.5%) | 5 (55.6%) | 5 (50.0%) | 2 (28.6%) |
| Female | 4 (44.4%) | 7 (87.5%) | 4 (44.4%) | 5 (50.0%) | 5 (71.4%) |
| Batch |  |  |  |  |  |
| 1 | 2 (22.2%) | 0 | 0 | 2 (20.0%) | 0 |
| 2 | 0 | 1 (12.5%) | 1 (11.1%) | 3 (30.0%) | 2 (28.5%) |
| 3 | 3 (33.3%) | 6 (75.0%) | 8 (88.9%) | 5 (50.0%) | 4 (57.1%) |
| 4 | 4 (44.4%) | 1 (12.5%) | 0 | 0 | 1 (14.3%) |
