# Supplementary material for: Bioinformatics and system biology approach to identify the influences of SARS-CoV-2 on metabolic unhealthy obese patients
Source: Front Mol Biosci. 2023 Oct 9;10:1274463. doi: 10.3389/fmolb.2023.1274463 (PMC10591333; doi:10.3389/fmolb.2023.1274463)
Supplement: Supplementary file 4 [file Table7.DOCX]

**Supplementary Table 7.** Top 10 TFs in network.

| Rank | Name | Description | Function | Reference |
| --- | --- | --- | --- | --- |
| 1 | FOXC1 | Forkhead Box C1 | Playing a role in the regulation of embryonic and ocular development | [1] |
| 2 | GATA2 | GATA Binding Protein 2 | Playing an essential role in regulating transcription of genes involved in the development and proliferation of hematopoietic and endocrine cell lineages | [2] |
| 3 | YY1 | YY1 Transcription Facto | Directing histone deacetylases and histone acetyltransferases to a promoter in order to activate or repress the promoter, thus implicating histone modification in the function of YY1 | [3] |
| 4 | DHRS9 | Dehydrogenase/Reductase 9 | Demonstrating oxidoreductase activity toward hydroxysteroids and is able to convert 3-alpha-tetrahydroprogesterone to dihydroxyprogesterone and 3-alpha-androstanediol to dihydroxyprogesterone in the cytoplasm | [4] |
| 5 | NFIC | Nuclear Factor I C | Function as cellular transcription factors and as replication factors for adenovirus DNA replication | [5] |
| 6 | CREB1 | CAMP Responsive Element Binding Protein 1 | Binding as a homodimer to the cAMP-responsive element, an octameric palindrome | [6] |
| 7 | JUN | Jun Proto-Oncogene, AP-1 Transcription Factor Subunit | Interacting directly with specific target DNA sequences to regulate gene expression | [7] |
| 8 | TMC5 | Transmembrane Channel Like 5 | Enable mechanosensitive ion channel activity | [8] |
| 9 | HINFP | Histone H4 Transcription Factor | Playing a role in DNA methylation and transcription repression | [9] |
| 10 | FOXL1 | Forkhead Box L1 | Playing critical roles in the regulation of multiple processes including metabolism, cell proliferation and gene expression during ontogenesis | [8] |

1. Göös, H.; Kinnunen, M.; Salokas, K.; Tan, Z.; Liu, X.; Yadav, L.; Zhang, Q.; Wei, G.H.; Varjosalo, M. Human transcription factor protein interaction networks. *Nat Commun* **2022**, *13*, 766, doi:10.1038/s41467-022-28341-5.

2. Huttlin, E.L.; Bruckner, R.J.; Paulo, J.A.; Cannon, J.R.; Ting, L.; Baltier, K.; Colby, G.; Gebreab, F.; Gygi, M.P.; Parzen, H.; et al. Architecture of the human interactome defines protein communities and disease networks. *Nature* **2017**, *545*, 505-509, doi:10.1038/nature22366.

3. Vaquerizas, J.M.; Kummerfeld, S.K.; Teichmann, S.A.; Luscombe, N.M. A census of human transcription factors: function, expression and evolution. *Nat Rev Genet* **2009**, *10*, 252-263, doi:10.1038/nrg2538.

4. Huttlin, E.L.; Ting, L.; Bruckner, R.J.; Gebreab, F.; Gygi, M.P.; Szpyt, J.; Tam, S.; Zarraga, G.; Colby, G.; Baltier, K.; et al. The BioPlex Network: A Systematic Exploration of the Human Interactome. *Cell* **2015**, *162*, 425-440, doi:10.1016/j.cell.2015.06.043.

5. Olsen, J.V.; Vermeulen, M.; Santamaria, A.; Kumar, C.; Miller, M.L.; Jensen, L.J.; Gnad, F.; Cox, J.; Jensen, T.S.; Nigg, E.A.; et al. Quantitative phosphoproteomics reveals widespread full phosphorylation site occupancy during mitosis. *Sci Signal* **2010**, *3*, ra3, doi:10.1126/scisignal.2000475.

6. Matsuoka, S.; Ballif, B.A.; Smogorzewska, A.; McDonald, E.R., 3rd; Hurov, K.E.; Luo, J.; Bakalarski, C.E.; Zhao, Z.; Solimini, N.; Lerenthal, Y.; et al. ATM and ATR substrate analysis reveals extensive protein networks responsive to DNA damage. *Science* **2007**, *316*, 1160-1166, doi:10.1126/science.1140321.

7. Niehrs, C. The complex world of WNT receptor signalling. *Nat Rev Mol Cell Biol* **2012**, *13*, 767-779, doi:10.1038/nrm3470.

8. Gaudet, P.; Livstone, M.S.; Lewis, S.E.; Thomas, P.D. Phylogenetic-based propagation of functional annotations within the Gene Ontology consortium. *Brief Bioinform* **2011**, *12*, 449-462, doi:10.1093/bib/bbr042.

9. Castello, A.; Fischer, B.; Eichelbaum, K.; Horos, R.; Beckmann, B.M.; Strein, C.; Davey, N.E.; Humphreys, D.T.; Preiss, T.; Steinmetz, L.M.; et al. Insights into RNA biology from an atlas of mammalian mRNA-binding proteins. *Cell* **2012**, *149*, 1393-1406, doi:10.1016/j.cell.2012.04.031.
