# Supplementary material for: Bioinformatics and system biology approach to identify the influences of SARS-CoV-2 on metabolic unhealthy obese patients
Source: Front Mol Biosci. 2023 Oct 9;10:1274463. doi: 10.3389/fmolb.2023.1274463 (PMC10591333; doi:10.3389/fmolb.2023.1274463)
Supplement: Supplementary file 6 [file Table6.DOCX]

**Supplementary Table 6.** Top 10 hub genes in network ranked by MCC method.

| Rank | Name | Description | Type | Function | Reference |
| --- | --- | --- | --- | --- | --- |
| 1 | SPI1 | Spi-1 proto-oncogene | Protein Coding | An ETS-domain transcription factor that activates gene expression during myeloid and B-lymphoid cell development | [1] |
| 2 | CD163 | CD163 Molecule | Protein Coding | An acute phase-regulated receptor involved in the clearance and endocytosis of hemoglobin/haptoglobin complexes by macrophages | [2] |
| 3 | C1QB | Complement C1q B Chain | Protein Coding | The B-chain polypeptide of serum complement subcomponent C1q, which associates with C1r and C1s to yield the first component of the serum complement system | [3] |
| 4 | SIGLEC1 | Sialic Acid Binding Ig Like Lectin 1 | Protein Coding | A lectin-like adhesion molecule that binds glycoconjugate ligands on cell surfaces in a sialic acid-dependent manner | [4] |
| 5 | C1QA | Complement C1q A Chain | Protein Coding | The A-chain polypeptide of serum complement subcomponent C1q, which associates with C1r and C1s to yield the first component of the serum complement system | [3] |
| 6 | ITGAM | Integrin Subunit Alpha M | Protein Coding | Regulating the adherence of neutrophils and monocytes to stimulated endothelium, and also in the phagocytosis of complement coated particles | [5] |
| 7 | CD14 | CD14 Molecule | Protein Coding | A surface antigen that is preferentially expressed on monocytes/macrophages | [6] |
| 8 | FCGR1A | Fc Gamma Receptor Ia | Protein Coding | A high-affinity Fc-gamma receptor | [7] |
| 9 | VSIG4 | V-Set And Immunoglobulin Domain Containing 4 | Protein Coding | A negative regulator of T-cell responses | [8] |
| 10 | C1QC | Complement C1q C Chain | Protein Coding | The C-chain polypeptide of serum complement subcomponent C1q, which associates with C1r and C1s to yield the first component of the serum complement system | [3] |

1. Wang, X.; Jin, P.; Zhang, Y.; Wang, K. CircSPI1 acts as an oncogene in acute myeloid leukemia through antagonizing SPI1 and interacting with microRNAs. *Cell Death Dis* **2021**, *12*, 297, doi:10.1038/s41419-021-03566-2.

2. Buechler, C.; Ritter, M.; Orsó, E.; Langmann, T.; Klucken, J.; Schmitz, G. Regulation of scavenger receptor CD163 expression in human monocytes and macrophages by pro- and antiinflammatory stimuli. *J Leukoc Biol* **2000**, *67*, 97-103.

3. Gaboriaud, C.; Juanhuix, J.; Gruez, A.; Lacroix, M.; Darnault, C.; Pignol, D.; Verger, D.; Fontecilla-Camps, J.C.; Arlaud, G.J. The crystal structure of the globular head of complement protein C1q provides a basis for its versatile recognition properties. *J Biol Chem* **2003**, *278*, 46974-46982, doi:10.1074/jbc.M307764200.

4. Izquierdo-Useros, N.; Lorizate, M.; Puertas, M.C.; Rodriguez-Plata, M.T.; Zangger, N.; Erikson, E.; Pino, M.; Erkizia, I.; Glass, B.; Clotet, B.; et al. Siglec-1 is a novel dendritic cell receptor that mediates HIV-1 trans-infection through recognition of viral membrane gangliosides. *PLoS Biol* **2012**, *10*, e1001448, doi:10.1371/journal.pbio.1001448.

5. Bai, M.; Grieshaber-Bouyer, R.; Wang, J.; Schmider, A.B.; Wilson, Z.S.; Zeng, L.; Halyabar, O.; Godin, M.D.; Nguyen, H.N.; Levescot, A.; et al. CD177 modulates human neutrophil migration through activation-mediated integrin and chemoreceptor regulation. *Blood* **2017**, *130*, 2092-2100, doi:10.1182/blood-2017-03-768507.

6. Cros, J.; Cagnard, N.; Woollard, K.; Patey, N.; Zhang, S.Y.; Senechal, B.; Puel, A.; Biswas, S.K.; Moshous, D.; Picard, C.; et al. Human CD14dim monocytes patrol and sense nucleic acids and viruses via TLR7 and TLR8 receptors. *Immunity* **2010**, *33*, 375-386, doi:10.1016/j.immuni.2010.08.012.

7. Guilliams, M.; Bruhns, P.; Saeys, Y.; Hammad, H.; Lambrecht, B.N. The function of Fcγ receptors in dendritic cells and macrophages. *Nat Rev Immunol* **2014**, *14*, 94-108, doi:10.1038/nri3582.

8. Vogt, L.; Schmitz, N.; Kurrer, M.O.; Bauer, M.; Hinton, H.I.; Behnke, S.; Gatto, D.; Sebbel, P.; Beerli, R.R.; Sonderegger, I.; et al. VSIG4, a B7 family-related protein, is a negative regulator of T cell activation. *J Clin Invest* **2006**, *116*, 2817-2826, doi:10.1172/jci25673.
